# The impact of health literacy on quality of life in patients with chronic diseases

**DOI:** 10.3389/fpubh.2025.1544259

**Published:** 2025-06-04

**Authors:** Jiali Zhang, Yinhai Chen, Yuanwei Lu, Xuan Jiang, Congxuan Lin, Xiong Ke

**Affiliations:** ^1^School of Public Health, North Sichuan Medical College, Nanchong, China; ^2^School of Nursing, North Sichuan Medical College, Nanchong, China; ^3^School of Management, North Sichuan Medical College, Nanchong, China; ^4^Sichuan Primary Health Research Center, North Sichuan Medical College, Nanchong, China; ^5^Key Laboratory of Digital-Intelligent Disease Surveillance and Health Governance, North Sichuan Medical College, Nanchong, China

**Keywords:** health literacy, quality of life, chronic disease patients, association, relationship

## Abstract

**Objective:**

We aimed to assess quality of life in patients with chronic diseases and identify influencing factors, as well as to explore the relationship between health literacy and quality of life in this population.

**Methods:**

We used health literacy and EuroQol 5-Dimensions 5-Level version survey data from chronically ill patients in the 2023 Sichuan Province Chinese Resident Psychological and Behavioral Survey Study. We analyzed factors influencing quality of life using a tobit regression model and examined the relationship between quality of life and health literacy using canonical correlation analysis.

**Results:**

The health utility value for quality of life among 611 patients with chronic diseases was 0.95 (0.86–1), with an EuroQol Visual Analog Scale score of 71.04 ± 16.21. Regression analysis revealed that health literacy (*p* = 0.004), sex (*p* = 0.015), body mass index (*p* = 0.047), occupation (*p* = 0.012), marital status (*p* = 0.026), debt status (*p* = 0.001), comorbidity (*p* < 0.001) and living alone (*p* = 0.033) were significantly associated with quality of life. Canonical correlation analysis showed a correlation of 0.269 (*p* < 0.001) between health literacy and quality of life, primarily related to factors such as treatment information, mental health, and vaccine type, which were correlated with pain or discomfort.

**Conclusion:**

Enhancing health literacy can positively impact the life quality of patients with chronic diseases. Key elements of health literacy interventions should include evaluating treatment information, accessing resources to address mental health concerns, and determining individual vaccine needs. Health education strategies should be developed to improve both health literacy and quality of life for patients with chronic diseases.

## 1 Introduction

With an aging population and changes in lifestyle and dietary habits, chronic diseases (primarily cardiovascular, cerebrovascular, and respiratory diseases; diabetes mellitus; and hypertension) have become major public health challenges in China (1). Chronic diseases are typically long-lasting, and patients are susceptible to various complications that can substantially impact their physical and mental health as well as their quality of life (QoL) (2). Approximately one-third of adults worldwide currently have comorbid chronic diseases, with even higher rates among older adults. In China, the prevalence of non-communicable diseases has increased sharply, from 17.0% in 1993 to 34.3% in 2018, with further growth expected (3, 4). Chronic diseases are associated with a wide range of health challenges, contributing to conditions such as depression (5), anxiety (6), and cognitive dysfunction (7); these diseases are also linked to a decline in QoL (8). Therefore, it is crucial to investigate QoL and its influencing factors in patients with chronic diseases and to implement targeted prevention and management strategies to mitigate the adverse effects of these conditions (9).

QoL refers to an individual’s subjective perception of physical function, their psychological state, and their social abilities within the framework of their values and cultural system (10). Despite rapid socioeconomic development, morbidity and mortality associated with chronic diseases remain high, and previous studies have demonstrated a decline in QoL among patients with chronic diseases, to varying extents (8). Improving QoL among patients with chronic conditions has therefore become a global health priority (11).

Multiple factors influence QoL, including an individual’s personality traits (12), anxiety and depression (13), social support (14), disease perception (15), sleep disorders (16), age (17), marital status (18), and ethnicity (19). In addition, research indicates that health literacy can substantially impact QoL. Health literacy refers to an individual’s ability to access, comprehend, and use health information to make informed health decisions, thereby maintaining or enhancing their QoL (20). Patients with higher health literacy levels tend to exhibit better self-care behaviors. Health literacy is crucial to the prevention, management, and treatment outcomes of chronic diseases (21). However, health literacy is often low among patients with chronic diseases, which may contribute to poorer health outcomes, limited self-management skills, and an increased mortality risk (22). Health literacy is shaped by factors such as education, socioeconomic status, and cultural background, making it a key health determinant (23).

Currently, the relationship between health literacy and QoL remains uncertain. In a cross-sectional study of Korean adults, Song found that low health literacy was a risk factor for poor health outcomes and lower health-related QoL (24). Mehralian demonstrated that health literacy levels among older patients in southern Iran were directly and significantly correlated with QoL at discharge (25). Naimi et al. reported a positive association between health literacy and QoL among patients with hypertension (26). A study by Aryankhesal among 175 older adult residents of nursing homes revealed that health literacy had a predictive power of 31.98% on QoL (27). However, some studies have reported contradictory findings. Ahmadzadeh et al. conducted a cross-sectional study involving 200 patients with heart failure in Iran and found no statistically significant association between health literacy and QoL (28). Yehle showed that enhancing health literacy among patients with heart failure via health education did not impact health-related QoL (29). Similarly, a cohort study by Montbleau et al. demonstrated no relationship between health literacy and QoL in patients with atrial fibrillation (30). Lee investigated patients with type 2 diabetes and revealed that health literacy only had an indirect effect on QoL (31). In China, research on the intrinsic relationship between health literacy and QoL among patients with chronic diseases is limited.

In this study, we aimed to assess QoL among Chinese patients with chronic diseases, analyze its influencing factors, and use Canonical Correlation Analysis (CCA) to explore the multidimensional relationships between health literacy and QoL. The findings aim to provide valuable insights for enhancing QoL in this patient population.

## 2 Methods

### 2.1 Study design and participants

This study used a cross-sectional design, jointly initiated by the School of Public Health of Peking University and other institutions, and drawing on health literacy as well as QoL data from the 2023 Survey of Chinese Residents’ Psychology and Behavior (PBICR) for patients with chronic diseases in Sichuan Province. Ethical approval was obtained from Shandong Provincial Hospital (SWYX: No. 2023-198), and informed consent was secured from all of the participants. The inclusion criteria were: (1) age 18 years or older; (2) able to complete an online questionnaire independently or with assistance. The exclusion criteria were: (1) delirium or mental disorders; (2) participation in other similar studies or prior participation in the PBICR.

### 2.2 Survey population

Based on the population demographics of Sichuan Province, we randomly selected 12 cities using a random number table method. In each city, six rural and four urban communities were chosen; residents were sampled via quota within each community. After applying the inclusion and exclusion criteria, the study ultimately included clinical data from 611 eligible patients with chronic diseases for analysis.

### 2.3 Survey tool

#### 2.3.1 General demographic characteristics and health status

We collected information on sex, ethnicity, body mass index (BMI), occupational status, education level, age, household registration, marital status, debt, per capita income, drinking, smoking, and comorbidity (defined as the condition of having two or more chronic diseases at the same time), for a total of 13 items.

#### 2.3.2 Health literacy scale

Health literacy was assessed using the short form of the Health Literacy Scale (HLS-SF), developed by Duong et al. (32). This four-item questionnaire, known as the HLS-SF4, is designed for the Chinese population is a reliable and valid tool (33). The HLS-SF4 uses a 4-point Likert scale (1 = very difficult, 2 = difficult, 3 = easy, 4 = very easy) to obtain a standardized health literacy index (HL index), which ranges from 0 to 50. Higher scores indicate greater health literacy. The calculation formula is HL Index = (Mean − 1) × (50/3) (33). In this study, the Cronbach’s α coefficient for the scale was 0.878, indicating good reliability and validity.

#### 2.3.3 Quality of life scale

QoL was measured using the EuroQol 5-Dimensions 5-Level version (EQ-5D-5L) scale, which includes two components: a self-assessment health status questionnaire and the EuroQol visual analogue scale (EQ-VAS) (34). The self-assessment questionnaire includes five dimensions, namely, mobility, self-care, daily activities, pain/discomfort, and anxiety/depression. Each of the five dimensions has five levels of difficulty: no difficulty, slight difficulty, moderate difficulty, severe difficulty, and extreme difficulty; these levels are represented using scores from 1 to 5, respectively (35). The health status in the five dimensions of the scale is coded, with a total of 3,125 potential health states, where “11,111” represents no difficulties in any dimension (full health), and “55,555” represents a state of extreme difficulty (36). In this study, we used the EQ-5D-5L utility scoring system, calibrated for the Chinese population, to convert health status into a health utility value ranging from −0.391to 1, with higher values indicating better health (37). The VAS rating was used to assess respondents’ self-reported health status, using a scale from 0 to 100, with higher scores indicating better self-perceived health status. The Cronbach’s α coefficient in this study was 0.835, indicating strong reliability and validity.

### 2.4 Quality control

All of the questionnaires were developed after an extensive review of the relevant literature and resources. Prior to administering the study, experts reviewed the questionnaire, and a pre-survey was conducted. Feedback from respondents was promptly collected, organized, and reviewed by the research team, and adjustments were made as needed. After standardized training, all of the investigators administered the survey, following the established protocol. Upon completion, two researchers independently conducted logical checks and screened the data.

### 2.5 Statistical analysis

The collected data were organized in Excel 365.0, with primary statistical analyses conducted using IBM SPSS 27.0 and tobit regression analysis carried out in Stata 18.0. Categorical data are presented as frequency and percentage. Because the health utility data did not follow a normal distribution, the median and interquartile range (P25, P75) were used, and the Mann–Whitney U test or Kruskal–Wallis H rank-sum test was applied to compare group differences in Qol. Considering the double—bounded nature of QoL (ranging from −0.391 to 1) and that the residuals conformed to a normal distribution. After standardizing the health literacy, the Tobit regression method was applied to analyze factors influencing QoL in patients with chronic diseases, and CCA was used to explore the relationship between health literacy and QoL. The statistical significance level was set to *p* < 0.05.

CCA is a multivariate statistical method that can help in examining the linear interrelationships between two sets of variables (38). This method uses dimensionality reduction to extract two representative integrated random variables (canonical variables) from two sets of variables. By maximizing the correlation between linear combinations of the two sets of variables, CCA can reveal the potential associations among multidimensional data (39).

## 3 Results

### 3.1 Sample characteristics

Among the 611 participants, health literacy scores demonstrated a mean of 22.65 ± 11.00 (range: 0–50), while QoL measurements showed a mean score of 0.89 ± 0.16 (observed range: −0.11 to 1.00). The majority had Han nationality (93.62%), and approximately half were women (52.37%). Most participants were within the normal BMI range, and 43.37% were employed. Educational levels varied, with 38.63% having completed primary school or below and 39.44% having attained a high school education level or above. A significant portion were over 60 years old (45.66%) and held agricultural household registrations (66.12%). In addition, 74.96% were married, 82.16% lived alone, and 72.34% were debt-free. Approximately half (52.37%) had a per capita income of less than 3,000 RMB. Non-drinking and non-smoking individuals constituted 62.68% and 78.4% of the sample, respectively, and 60.07% of the patients had no comorbidity; among comorbidity, cardiovascular diseases accounted for the largest proportion (21.28%)(Table 1).

**Table 1:** Statistical characteristics of participants.

Item	Group	Frequency (%)
Sex	Female	320 (52.37)
	Male	291 (47.63)
Ethnicity	Han nationality	572 (93.62)
	Minority	39 (6.38)
BMI	Underweight	59 (9.66)
	Normal	408 (66.78)
	Overweight or obese	144 (23.57)
Occupation	Employed	265 (43.37)
	Student	34 (5.56)
	Retired	189 (30.93)
	Unemployed	123 (20.13)
Education	Primary school or below	236 (38.63)
	Junior high school	134 (21.93)
	High school or above	241 (39.44)
Age(years)	<30	65 (10.64)
	30–44	84 (13.75)
	45–59	183 (29.95)
	≥60	279 (45.66)
Household registration	Non-rural	207 (33.88)
	Rural	404 (66.12)
Marital status	Unmarried	65 (10.64)
	Married	458 (74.96)
	Other	88 (14.40)
Living alone	No	502 (82.16)
	Yes	109 (17.84)
Debt	No	442 (72.34)
	Yes	169 (27.66)
Per capita income(RMB)	≤3,000	320 (52.37)
	3,001–6,000	199 (32.57)
	>6,000	92 (15.06)
Drinking	No	383 (62.68)
	Quit drinking	89 (14.57)
	Yes	139 (22.75)
Smoking	No	479 (78.40)
	Quit smoking	45 (7.36)
	Yes	87 (14.24)
Chronic disease types	Comorbidity^1^	244 (39.93)
	Cardiovascular system	130 (21.28)
	Other diseases	94 (15.38)
	Digestive system	39 (6.38)
	Musculoskeletal system	37 (6.06)
	Endocrine system	25 (4.09)
	Respiratory system	21 (3.44)
	Tumors	13 (2.13)
	Urinary system	8 (1.31)

1 Comorbidity: The condition of having two or more chronic diseases at the same time.

### 3.2 Life quality level

**Figure 1:**
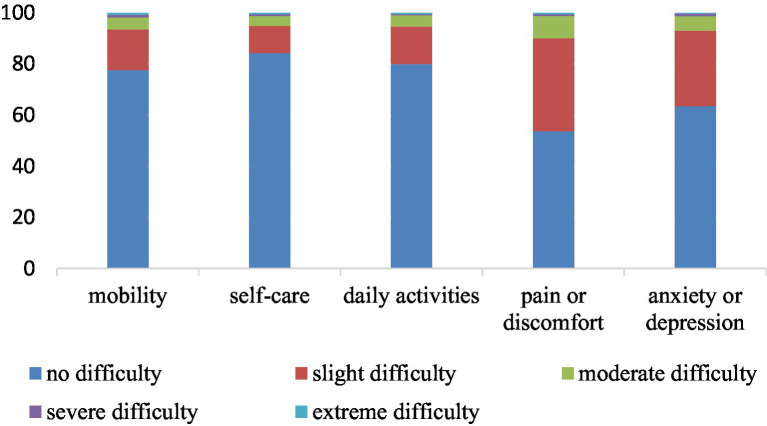
Dimensions distribution of EQ-5D-5L.

The mean EQ-VAS score of patients with chronic diseases in the study area was (71.04 ± 16.21). The proportion of patients with difficulty in the five dimensions of mobility, self-care, daily activities, pain or discomfort, and anxiety or depression was 22.4%, 15.7%, 20%, 46.3%, and 36.5%, respectively (Figure 1).

### 3.3 Single-factor analysis of QoL

In this study, we conducted univariate analysis for QoL among patients with chronic diseases. The results showed that sex, ethnicity, BMI, occupational status, educational level, age, household registration, marital status, living alone, per capita income, drinking, and comorbidity were statistically significant in the comparison of QoL (*p* < 0.05), as shown in Table 2.

**Table 2:** Single factor analysis of quality of life.^1^

Item	Group	QoL	Z/H	*p*-value
Sex	Female	0.94 (0.83 ~ 1.00)	−2.587	0.01
	Male	1.00 (0.88 ~ 1.00)		
Ethnicity	Han	0.95 (0.88 ~ 1.00)	−3.254	0.001
	Minority	0.89 (0.64 ~ 0.95)		
BMI	Underweight	0.94 (0.79 ~ 0.95)	7.824	0.02
	Normal	0.95 (0.83 ~ 1.00)		
	Overweight or obese	0.95 (0.89 ~ 1.00)		
Occupation	Employed	0.95 (0.89 ~ 1.00)	16.153	0.001
	Student	0.95 (0.89 ~ 1.00)		
	Retired	0.94 (0.73 ~ 1.00)		
	Unemployed	0.94 (0.78 ~ 1.00)		
Education	Primary or below	0.94 (0.81 ~ 1.00)	15.545	<0.001
	Junior high school	1.00 (0.89 ~ 1.00)		
	High school or above	0.95 (0.88 ~ 1.00)		
Age(years)	<30	0.95 (0.89 ~ 1.00)	24.833	<0.001
	30–44	0.98 (0.89 ~ 1.00)		
	45–59	1.00 (0.89 ~ 1.00)		
	≥60	0.94 (0.76 ~ 1.00)		
Household registration	Non-rural	1.00 (0.89 ~ 1.00)	−4.601	<0.001
	Rural	0.94 (0.81 ~ 1.00)		
Marital status	Unmarried	0.94 (0.88 ~ 1.00)	17.764	<0.001
	Married	0.95 (0.88 ~ 1.00)		
	Other	0.89 (0.73 ~ 1.00)		
Living alone	No	0.94 (0.73 ~ 1.00)	−3.108	0.002
	Yes	0.95 (0.88 ~ 1.00)		
Debt	No	0.95 (0.86 ~ 1.00)	−1.33	0.184
	Yes	0.94 (0.87 ~ 1.00)		
Per capita income(RMB)	≤3,000	0.94 (0.78 ~ 1.00)	21.379	<0.001
	3,001–6,000	0.95 (0.88 ~ 1.00)		
	>6,000	1.00 (0.94 ~ 1.00)		
Drinking	No	0.94 (0.85 ~ 1.00)	8.595	0.014
	Quit drinking	0.90 (0.78 ~ 1.00)		
Smoking	Yes	1.00 (0.89 ~ 1.00)		
	No	0.94 (0.85 ~ 1.00)	2.118	0.347
	Quit smoking	0.94 (0.79 ~ 1.00)		
Comorbidity	Yes	0.95 (0.89 ~ 1.00)		
	No	1.00 (0.89 ~ 1.00)	−6.984	<0.001
	Yes	0.89 (0.73 ~ 1.00)		

1 Single factor analysis.

### 3.4 Spearman correlation analysis for health literacy and QoL

Among the 611 respondents, 53.7%, 63.1%, 63%, and 57.9% had difficulties in finding information on disease treatment, judging the advantages and disadvantages of treatment, coping with mental health problems, and judging the appropriate types of vaccines, respectively. Spearman correlation analysis was used in this study to further analyze the impact of health literacy on QoL. The results of statistical analysis showed that health literacy was positively correlated with QoL in patients with chronic diseases (r = 0.255, *p* < 0.01) and was correlated with multiple dimensions (Supplementary Table 1).

### 3.5 Tobit regression model analysis results of QoL

The QoL were analyzed as the dependent variable using a Tobit regression model with other covariates as independent variables. The model demonstrated statistically significant explanatory power, as evidenced by a likelihood ratio chi-square statistic of 153.21 (Prob > chi2 = 0.0000). Several variables were significantly associated with QoL in the final model: health literacy (*p* = 0.004), sex (*p* = 0.015), BMI (*p* = 0.047), occupation (*p* = 0.012), marital status (*p* = 0.026), debt (*p* = 0.001), comorbidity (*p* < 0.001) and living alone (*p* = 0.033) (Table 3).

**Table 3:** Tobit regression model analysis results of quality of life.^1^

Variable		Reference group	β	SE	P	95%CI
Constant			0.875	0.071	<0.001	(0.736, 1.013)
Sex	Male	Female	0.057	0.023	0.015	(0.011, 0.103)
Household registration	Non-rural	Rural	0.045	0.024	0.057	(−0.001, 0.092)
Ethnicity	Minority	Han	0.079	0.040	0.050	(0.000, 0.159)
BMI	Underweight	Normal	−0.067	0.034	0.047	(−0.133,-0.001)
	Overweight or obese		0.004	0.024	0.860	(−0.044, 0.052)
Occupation	Student	Employed	0.158	0.063	0.012	(0.035, 0.282)
	Retired		−0.034	0.030	0.257	(−0.094, 0.025)
	Unemployed		−0.024	0.031	0.442	(−0.084, 0.037)
Education	Junior high school	Primary or below	0.040	0.029	0.165	(−0.017, 0.097)
	High school or above		−0.023	0.029	0.430	(−0.081, 0.034)
Age (years)	30–44	<30	0.081	0.054	0.132	(−0.024, 0.187)
	45–59		0.037	0.053	0.490	(−0.068, 0.141)
	≥60		−0.002	0.056	0.977	(−0.112, 0.108)
Marital status	Unmarried	Married	−0.098	0.050	0.050	(−0.195, 0.000)
	Other		−0.067	0.030	0.026	(−0.125,-0.008)
Per capita income (RMB)	3,001–6,000	≤3,000	0.042	0.023	0.072	(−0.004, 0.088)
	>6,000		0.043	0.034	0.207	(−0.024, 0.110)
Debt	Yes	No	−0.088	0.026	0.001	(−0.138,-0.037)
Comorbidity	Yes	No	−0.112	0.022	<0.001	(−0.155,-0.070)
Living alone	Yes	No	0.059	0.028	0.033	(0.005, 0.114)
Drinking	Quit drinking	No	−0.046	0.030	0.126	(−0.105, 0.013)
	Yes		0.038	0.029	0.194	(−0.019, 0.096)
Smoking	Quit smoking	No	−0.036	0.040	0.375	(−0.114, 0.043)
	Yes		−0.034	0.034	0.326	(−0.100, 0.033)
Health literacy			0.033	0.011	0.004	(0.011, 0.056)

1 Tobit regression.

### 3.6 CCA between health literacy and QoL

#### 3.6.1 CCA and significance test between health literacy and QoL

We analyzed the X variable set comprising four dimensions of health literacy (treatment information X1, treatment plan X2, mental health X3, and vaccine type X4) and the Y variable set comprising five dimensions of QoL (mobility Y1, self-care Y2, daily activities Y3, pain/discomfort Y4, and anxiety/depression Y5). In total, four groups of typical variables were obtained. Among these, the correlation coefficient of the first group of typical variables was 0.269 (*p* < 0.001); the first group of typical variables could explain 34.4% of the variation in the QoL variable and 58.4% of the variation in the health literacy variable (Supplementary Table 2).

#### 3.6.2 Typical coefficient of standardization for health literacy and QoL

In the first canonical variable health literacy (V1), the standardized canonical coefficients of treatment information, treatment plan, mental health, and vaccine type were 0.284, −0.262, −0.210, and −0.814, respectively, indicating that the type of vaccine needed had the greatest impact on health literacy. In the first typical variable QoL (W1), the standardized coefficients of mobility, self-care, daily activities, pain/discomfort, and anxiety/depression were 0.081, −0.567, 0.429, 0.937, 0.025, respectively; pain/discomfort was the main influencing variable (Table 4). According to the standardized canonical correlation coefficient, the linear combination within the canonical variable group is obtained as follows:


V1=0.284X1−0.262X2−0.210X3−0.814X4

W1=0.081Y1−0.567Y2+0.429Y3+0.937Y4+0.025Y5;

**Table 4:** Typical coefficients of health literacy standardization.

Original variable	Standardized canonical coefficient (V1)	Original variable	Standardized canonical coefficient (W1)
Treatment information (X1)	0.284	Mobility (Y1)	0.081
Treatment plan (X2)	−0.262	Self-care (Y2)	−0.567
Mental health issues (X3)	−0.210	Daily activities (Y3)	0.429
Vaccine type (X4)	−0.814	Pain or discomfort (Y4)	0.937
		Anxiety or depression (Y5)	0.025

Because each dimension of QoL is designed in reverse, it can be seen from the first linear combination that the canonical variables mainly demonstrated a positive correlation between type of vaccine (X4) and pain or discomfort (Y4).

#### 3.6.3 Analysis of typical structure of health literacy and QoL

**Figure 2:**
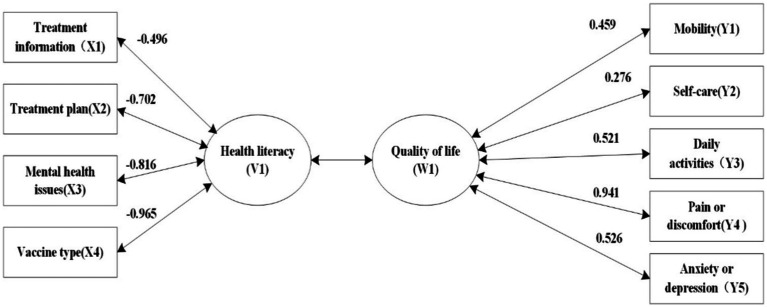
Typical load coefficient.

**Figure 3:**
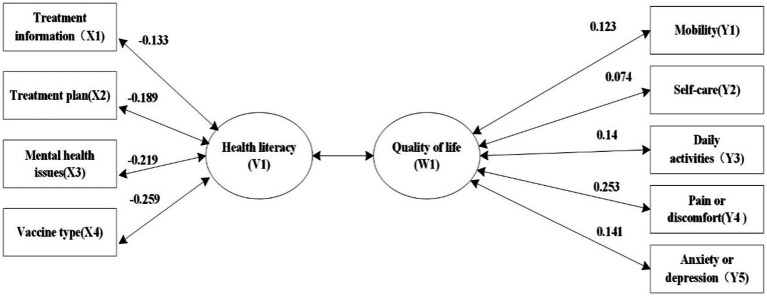
Typical cross-load coefficient.

To better reflect the relationship between the original variable and typical variable, we further analyzed the typical load and cross-load coefficients. According to the results of typical structural analysis, the typical variable of QoL W1 was most closely correlated with pain and discomfort (0.941). Health literacy V1 was mainly affected by treatment regimen (−0.702), mental health (−0.816), and judging the type of vaccine needed (−0.965). Additionally, as shown in Figures 2, 3, each item of health literacy is negatively related to V1, while each item of quality of life is positively related to U1. However, since quality of life is measured with reverse scoring, the data in Figures 2, 3 display a negative correlation. In reality, health literacy is positively correlated with the overall score and individual dimensions of quality of life.

## 4 Discussion

### 4.1 Quality of life needs to be improved in patients with chronic diseases

Our study showed that the value of health utility among patients with chronic diseases was 0.95 (0.86–1), in this study, consistent with findings from previous research among patients with chronic diseases (40). Our study targeted adults with chronic conditions who generally experience fewer difficulties in mobility, self-care, and daily activities; however, we found that their overall QoL still needed improvement. Patients with long-term diseases who have declining body functions and pain are prone to anxiety and depression. Studies show a 50% prevalence of anxiety and depression among these patients (41, 42). Most people experience some health-related anxiety during their lifetime, yet excessive levels can weaken the immune system, impair health-related decisions, and alter social behaviors, causing adverse physical reactions and further lowering their life quality (43). Thus, addressing anxiety and depression in patients with chronic diseases and providing timely psychological intervention and treatment is crucial for improving their overall health and QoL.

### 4.2 Factors influencing QoL in patients with chronic diseases

#### 4.2.1 Living alone is negatively correlated with QoL

The negative impact of living alone on QoL in patients with chronic diseases is consistent with the findings of Hu et al. (44). Patients who live alone often lack emotional support, which can lead to feelings of isolation and increase the risk of anxiety and depression (45). In daily life, the absence of assistance creates difficulties, including with disease self-management, and reduces medication adherence. Studies have found that QoL scores among people who live alone are usually lower than those who live with others, and that living alone significantly increases the risk of lower QoL (46). Additionally, patients who live alone may not make full use of medical resources, leading to a lack of timely monitoring and treatment of their conditions and an increased risk of deteriorating health (47). Therefore, society and medical institutions should actively provide emotional support, practical assistance, and access to medical resources for patients who are living alone. Communities can provide services in which volunteers regularly visit these patients, and online health platforms can offer real-time question-and-answer as well as other services to improve patients’ QoL.

#### 4.2.2 Multimorbidity is negatively correlated with QoL

Our findings showed that patients with chronic disease comorbidity exhibited lower QoL, a trend observed in previous studies (48), with an increase in comorbidity significantly correlated with decreased QoL (49). The long-term nature of chronic diseases is often accompanied by a gradual decline of physical functioning, which leads to some degree of physical dysfunction (50), disease-related pain (51), and sleep disorders (52), all of which can have a negative impact on QoL (53). These health problems may lead to more psychological pressure and emotional distress, resulting in anxiety, depression (54), and other psychological problems that result in fluctuating QoL levels (55). Multimorbidity can also increase the medical economic burden on patients and undermine their confidence in treatment. Individuals with inadequate health literacy are more likely to have a worse health condition. Health literacy plays a crucial role in an individual’s participation in health-related activities, medical decision-making, and disease prevention behaviors (56). Therefore, in clinical practice, greater attention is needed to the management of patients’ multimorbidity in which individualized treatment plans are developed and enhanced psychological support and health education are provided, so as to improve their QoL.

#### 4.2.3 Health literacy is positively correlated with life quality in patients with chronic diseases

Health literacy is a key factor in the QoL of patients with chronic diseases. Patients with lower health literacy often have trouble obtaining, understanding, and using health information. These patients tend to know less about their disease, have weaker self-management capabilities, and are less likely to adhere to treatment, all of which can affect their QoL. Research shows that health literacy has a positive impact on the QoL of patients with chronic diseases (57), which in turn is positively related to patients’ physical functioning, psychological state, and social skills. Moreover, health literacy affects how patients use medical and health resources. Individuals with low health literacy often cannot fully use these resources, leading to worse treatment results and lower QoL (58). Health care workers should improve health education for patients with lower health literacy to improve their self-management abilities and enhance their QoL.

### 4.3 CCA of health literacy and QoL

Enhancing health literacy can improve QoL, consistent with the findings of international research (59). In our study, CCA revealed that health literacy is positively correlated with all of the QoL dimensions among patients with chronic diseases. In the first set of canonical variables, treatment plan, mental health, and vaccine type showed high load coefficients; these are closely associated with daily activities, pain/discomfort, and anxiety/depression. Patients who are skilled at evaluating treatment options, determining their vaccine needs, and accessing mental health information tend to more effectively manage pain, discomfort, anxiety, and depression, in line with findings by Zhao et al. (60). Given the positive correlation between health literacy and QoL, targeted interventions should focus on educating patients with chronic diseases about disease risk, treatment planning, and preventive health measures to improve their health literacy, cultivate preventive health awareness, enhance preventive behaviors, and ultimately improve their QoL (61).

In this study, we used CCA to examine the intrinsic relationship between health literacy and QoL. We found that the ability to evaluate treatment options, locate mental health resources, and identify appropriate vaccines was strongly associated with daily activities, pain or discomfort, and anxiety or depression. Targeted improvements in health literacy in these areas may have implications for improving QoL in patients with chronic diseases. However, this study was cross-sectional, which reflects patient data at a single point in time; therefore, we cannot establish causality between health literacy and QoL in patients with chronic diseases. In addition, owing to logistical constraints, data were collected only from patients with chronic diseases in Sichuan Province, thus limiting the sample size and representativeness of the findings. Finally, it should be noted that our study overlooked the QoL of chronic disease types. Given the large number of chronic disease types involved, only the classification distribution of chronic disease types was presented in the demographic table, which might limit the clinical interpretability of the research results regarding the impact of disease heterogeneity on health outcomes.

## Data Availability

The original contributions presented in the study are included in the article/Supplementary material, further inquiries can be directed to the corresponding author.
