# Supplementary material for: The impact of health literacy on quality of life in patients with chronic diseases
Source: Front Public Health. 2025 Jun 4;13:1544259. doi: 10.3389/fpubh.2025.1544259 (PMC12186059; doi:10.3389/fpubh.2025.1544259)
Supplement: Supplementary file 1 [file Table_1.docx]

Supplementary Table 1

**Table 1 Correlation between health literacy and quality of life^[[1]](#footnote-0)^**

|  | HLS1 | HLS2 | HLS3 | HLS4 | Health literacy |
| --- | --- | --- | --- | --- | --- |
| Mobility | -0.197** | -0.190** | -0.146** | -0.147** | -0.207** |
| Self-care | -0.229** | -0.203** | -0.103* | -0.099* | -0.180** |
| Daily activities | -0.224** | -0.214** | -0.142** | -0.177** | -0.224** |
| Pain or discomfort | -0.175** | -0.208** | -0.225** | -0.238** | -0.250** |
| Anxiety or depression | -0.100* | -00.076 | -0.094* | -0.123** | -0.113** |
| Health utility value | 0.194** | 0.196** | 0.249** | 0.234** | 0.255** |

* P<0.05, ** P<0.01

1. Spearman correlation analysis [↑](#footnote-ref-0)
