# Supplementary material for: The impact of health literacy on quality of life in patients with chronic diseases
Source: Front Public Health. 2025 Jun 4;13:1544259. doi: 10.3389/fpubh.2025.1544259 (PMC12186059; doi:10.3389/fpubh.2025.1544259)
Supplement: Supplementary file 2 [file Table_2.docx]

Supplementary Table 2

**Table 2 Canonical correlation analysis between health literacy and quality of life variables**

| Canonical variable | Correlation | Wilk value | F-value | P-value |
| --- | --- | --- | --- | --- |
| 1 | 0.269 | 0.897 | 3.326 | <0.001 |
| 2 | 0.149 | 0.967 | 1.708 | 0.059 |
| 3 | 0.094 | 0.989 | 1.146 | 0.333 |
| 4 | 0.049 | 0.998 | - | - |
